# Athlete biological passport: longitudinal biomarkers and statistics in the fight against doping

**DOI:** 10.2478/aiht-2024-75-3793

**Published:** 2024-03-29

**Authors:** Dora Dragčević, Vlatka Pandžić Jakšić, Ozren Jakšić

**Affiliations:** University Hospital Merkur, Department of Haematology, Zagreb, Croatia; University Hospital Dubrava, Department of Endocrinology, Zagreb, Croatia; University of Zagreb School of Medicine, Zagreb, Croatia; University Hospital Dubrava, Department of Haematology, Zagreb, Croatia

**Keywords:** ABP, biochemical factors, confounding factors, haematological module, steroidal module, biokemijski čimbenici, biološka putovnica sportaša, hematološki modul, steroidni modul, zbunjujući čimbenici

## Abstract

As novel substances, short time windows, and limits of detection increasingly challenge direct methods of doping detection in sports, indirect tools inevitably take a greater role in the fight against it. One such tool is the athlete biological passport (ABP) – a longitudinal profiling of the measured haematological and biochemical biomarkers, combined with calculated scores, against the background of epidemiological data crucial for doping detection. In both of its modules, haematological and steroidal, ABP parameters are analysed with the Bayesian adaptive model, which individualises reference and cut-off values to improve its sensitivity. It takes into account the confounding factors with proven and potential influence on the biomarkers, such as race and altitude exposure. The ABP has already changed the fight against doping, but its importance will further grow with the new modules (e.g., endocrinological), parameters (e.g., plasma volume-independent parameters), and complementing indirect methods (e.g., transcriptomic).

## Abstract (hr)

Direktne metode detekcije dopinga u sportu neprestano se suočavaju s novim supstancijama, kratkim vremenskim okvirima i granicama mogućnosti detekcije, stoga su indirektne metode detekcije neizbježan dio suvremene borbe protiv dopinga. Referentne vrijednosti na razini populacije i univerzalne granice kao indirektne metode pokazale su nedostatnost, a multidisciplinarna istraživanja dovela su do implementacije Atletske biološke putovnice (ABP) s hematološkim i steroidnim modulom. Atletska biološka putovnica je longitudinalni profil mjerenih hematoloških i biokemijskih biomarkera – zajedno s izračunanim rezultatima, a u usporedbi s epidemiološkim podatcima koji su ključni za otkrivanje dopinga. U obama modulima ABP-a parametri se analiziraju Bayesovim adaptivnim modelom, što dovodi do personaliziranih referentnih i graničnih vrijednosti, a to poboljšava njegovu osjetljivost. Zbunjujući čimbenici s dokazanim i potencijalnim utjecajem na biomarkere (npr. etničko podrijetlo, izloženost nadmorskoj visini) također su važni u analizi ABP-a. Bez sumnje, ABP je promijenio borbu protiv dopinga. Razvijanje novih modula (npr. endokrinološki), novih parametara (npr. neovisnih o volumenu plazme) i dopuna neizravnih metoda (npr. transkriptomika) mogu dodatno povećati važnost ABP-a u budućnosti.

Doping, in general, is any abuse of illegal substances or methods to improve one’s performance and achieve desired results (1). In sports, the most common and widely known forms of doping are anabolic steroids and blood doping (boosting the red blood cell count) (2).

The aim of organised fight against doping is not only to ensure fair play but also to protect the health of athletes, because history has taught us that doping can end with fatalities (2, 3). Early on, anti-doping programmes relied solely on direct detection of specific compounds, but technological development enabling rapid synthesis of novel substances or implementation of novel doping techniques rendered those methods always lagging one step behind. The inadequacy of direct methods for doping detection reached the public with the Operación Puerto (Operation Mountain Pass) in 2006, when Spanish police found a great number of anabolic steroids, recombinant human erythropoietin (rHuEPO), preserved blood bags for autologous blood transfusion, and laboratory equipment and charged a number of athletes and their teams of doping abuse (3,4,5). This incident turned the attention to indirect detection methods designed to detect abnormal changes in biological parameters caused by doping. The main challenge of these methods is to recognise when the change in measured parameters is owed to doping and when it results from confounding factors such as physiological changes or illness. To meet this challenge, the World Anti-Doping Agency (WADA) as the head anti-doping organisation has developed a strict system called the athlete biological passport (ABP).

## ABP: MAIN IDEA AND IMPLEMENTATION

**Figure 1:**
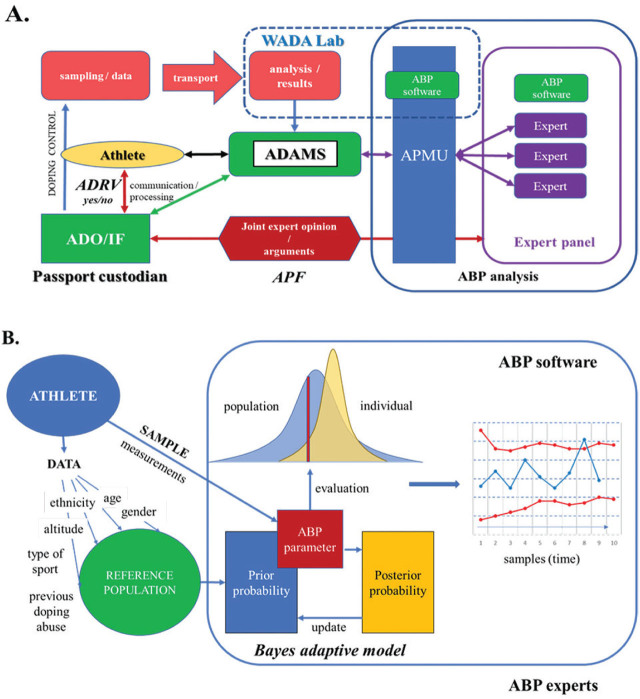
Athlete biological passport (ABP) flowchart process (A) and analyses (B). ABP – athlete biological passport, ADAMS – anti-doping administration and management system, ADO – anti-doping organisation, ADRV – anti-doping rule violation, APF – adverse passport finding, APMU – athlete’s passport management unit, IF – international federation, WADA – World Anti-Doping Agency

The ABP is an indirect method for doping detection, whose goal is to detect and red-flag changes in measured biological parameters resulting from doping abuse (e.g., rHuEPO) and distinguish them from those resulting from physiological changes (e.g., adaptation to altitude) (6, 7). This sophisticated indirect tool was preceded by comparing blood markers measured in athletes with universal population-based upper limits for haemoglobin (HGB; 175 g/L for men and 150 g/L for women), haematocrit (Hct; 50 % for men and 47 % for women), and reticulocyte percentage (Ret%; 2 % for both sexes) (4, 7), which could not account for confounding factors (e.g., altitude exposure, race) and for titrating doping doses so as not to cross these limits (8). For example, scientific research has shown that about 3.9 % of non-athlete males and 10.4 % of elite male rowers have physiological Hct values above 51 % (9). To avoid indicting clean athletes, the approach had to be refined. Scientific research in sports physiology, careful review of findings in doped and clean athletes, and statistical analyses eventually yielded a new tool in 2009 – the ABP (Figure 1), which was at first limited to its haematological module (10).

### Haematological module of the ABP

The main goal of blood doping (e.g., autologous blood transfusion, rHuEPO, hypoxia-inducible factors) is to raise haemoglobin levels and oxygen delivery to working muscles so as to delay anaerobic metabolism causing fatigue (11,12,13,14). As it may escape detection by direct methods, this ABP module is set to red-flag a non-physiological rise in blood parameters as suspicious. It consists of 12 measured parameters: Hct, HGB, immature reticulocyte fraction (IRF), red blood cell count (RBC#), Ret%, reticulocyte count (Ret#), mean corpuscular volume (MCV), mean corpuscular haemoglobin (MCH), mean corpuscular haemoglobin concentration (MCHC), platelet count (PLT), red blood cell distribution width (RDW), and white blood cell count (WBC). These parameters are used to determine two important indicators: the OFF-score and the abnormal blood profile score (ABPS) (Table 1) as described in detail elsewhere (15). Since the goal of the ABP is to individualise anti-doping testing, reference ranges of measured parameters are not only adapted statistically (as explained below) but are also adjusted according to the previous athlete’s data (longitudinal follow-up) and epidemiological characteristics (16,17,18,19,20,21).

**Table 1:** Measured and calculated parameters in athlete biological passport (ABP) modules

**ABP module**	**Measured parameters**	**Calculated parameters**
Haematological	HGB	
	Ret%	
	Hct	
	RBC#	OFF-score
	Ret#	$\text{OFFscore}=\left[\text{HGB}\right]\left[\frac{\text{g}}{\text{L}}\right]-60\sqrt{\text{Ret}\%}$
	IRF	)
	MCV	
	MCH	ABPS
	MCHC	Hct, HGB, RBC, Ret%, MCV, MCH, and MCHC
	RDW	
	WBC	
	PLT	

Steroid	testosterone	T/E
	epitestosterone	A/T
	etiocholanolone	A/Etio
	5α-androstanediol	5a-diol/5b-diol
	5β-androstandiol	

Endocrinological^*^	IGF-1	GH-2000
	P-III-NP	

* pending implementation. 5a-diol/5b-diol – 5α-androstanediol/5β-androstanediol ratio; A/Etio – androsterone/etiocholanolone ratio; A/T – androsterone/testosterone ratio; ABPS – abnormal blood profile score; GH-2000 – a calculated score from IGF-1 and P-III-NP serum concentrations corrected for sex and age; Hct – haematocrit; HGB – haemoglobin; IGH-1 – insulin-like growth factor 1; IRF – immature reticulocyte fraction; MCH – mean corpuscular haemoglobin; MCHC – mean corpuscular haemoglobin concentration; MCV – mean corpuscular volume; P-III-NP – procollagen III peptide; PLT – absolute platelet count; RBC – absolute red blood cell count; RDW – red blood cell distribution width; Ret% – reticulocyte percentage; Ret# – absolute reticulocyte count; T/E – testosterone/epitestosterone ratio; WBC – absolute white blood cell count

### Epidemiological characteristics important for the haematological module

Studies have verified that, regardless of race and age from 15 years onward, women have approximately 15–20 g/L lower HGB levels than men (22,23,24). In adolescent women, regardless of race, HGB values soar with puberty (at around 10–12 years of age), followed by a slight decrease and stabilisation from the age of 15 onwards (23, 25). On the other hand, HGB concentration in men rises continuously throughout the puberty to plateau at around 18 years of age (end of puberty) (23, 25). These constitutional differences between men and women are largely owed to androgen-stimulated and oestrogen-inhibited erythropoietin (EPO) secretion in the kidneys and to the combined stimulation of erythropoiesis in the bone marrow by EPO and androgens (24).

Differences may also arise from race. One study (26) reports that HGB and Hct are about 10 g/L and 2–3 % lower, respectively, in Africans and Asians than in Caucasians. One US study (27) reports significantly lower HGB, Hct, MCV, WBC, and PLT in African Americans than in the control Caucasian group, even after excluding individuals with alpha- and beta-thalassaemia or haemoglobin S. For example, it reports race differences for HGB of 7.2 g/L in women and 5.8 g/L in men, for Hct 1.55 % in women and 0.92 % in men, and for MCV 2.99 fL in women and 2.72 fL in men.

Other important epidemiological characteristics that may lead to differences in ABP parameters are the type of the sport and athlete’s altitude exposure. The success in aerobic sports correlates with maximal oxygen uptake volume (VO_2_ max), whereas in anaerobic sports the success is more dependent on lactate threshold and muscle properties (28). One study (29) has shown lower Hct and HGB, higher plasma volume, and higher total haemoglobin mass (tHbmass) in athletes in endurance (aerobic) than non-endurance (anaerobic) sports. Lower HGB and Hct are owed to a rise in the plasma volume because of hormonal response (e.g., aldosterone, growth hormone) to endurance training (29, 30).

Similarly, altitude exposure induces a rapid rise in HGB due to shifts in body fluids and a rise in tHbmass after 7–10 days due to EPO stimulation of the bone marrow and increased erythropoiesis (31). A meta-analysis by Lobigs et al. (31) evaluating how altitude exposure [or hypoxic dose, which is time spent at a certain altitude measured in kilometre hours (kmh)] affects HGB, Ret%, and OFF-score showed statistically significant changes in Ret% and OFF-score with altitude exposure ranging from 100–200 kmh and HGB plateauing at 9.4 g/L with altitude exposure of 1000 kmh. Upon return to the sea level, Ret% decrease correlates with the previous altitude exposure (higher decrease in individuals with 1500 kmh than in those with 500 kmh), OFF-score changes irregularly, and HGB levels return to baseline after two weeks regardless of the previous altitude exposure (31, 32).

### The statistics behind the ABP haematological module

**Figure 2:**
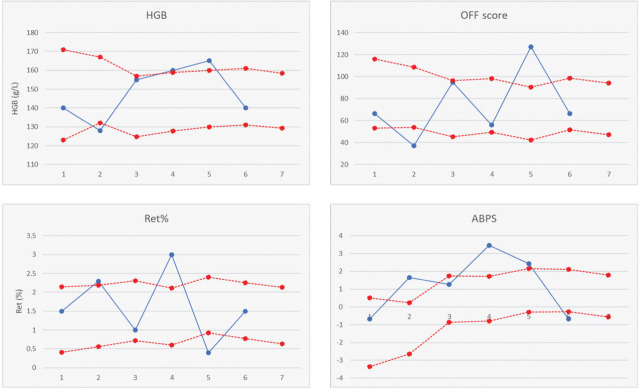
Examples of HGB, Ret%, OFF-score, and ABPS profiles in the athlete biological passport (ABP) indicative of different types of blood doping (based on available literature data about the influence of rHuEPO, blood transfusion, and blood withdrawal on erythropoiesis and ABP profiles). Legend: blue line – athlete’s measured data in each testing; red line – calculated (expected) reference ranges. Testing points: 1 – physiological values; 2 – withdrawal of 1 blood unit; 3 – reinfusion of 1 blood unit; 4 – beginning of rHuEPO application; 5 – rHuEPO cessation; 6 – physiological values; 7 – predicted reference range for the next testing

One statistical model that has proved greatly successful in doping detection regardless of the module is the Bayesian adaptive model, which calculates the probability of an event (e.g., doping) based on measured parameters, scientific data, expected intra- and inter-individual variability, and previous results (16,17,18,19, 33,34,35). For the haematological module, the Bayesian adaptive model considers four parameters: HGB, OFF-score, Ret%, and ABPS (Figure 2) (15). It also accounts for important confounding factors, including sex (male, female), age (<19 years, 19–24 years, >24 years), race (African, Asian, Caucasian, Oceanian), type of sport (endurance, non-endurance), altitude exposure (<610 m, 610–1730 m, >1730 m), and previous doping (doped, not doped) (21). With each new testing, the ABP software calculates the expected upper and lower reference range values in physiological conditions for an athlete (without a disease, not doped) (21). It marks suspicious changes with at least 99 % probability to minimise the rate of false positive results. It also groups the findings by probability as follows: 99.8–99.9 % practically proved doping, 99.1–99.79 % extremely likely doping, 95–99.09 % very likely, 90–94.99 % likely, 80–89.90 % undecided, and below 80 % not useful (21). Furthermore, if the OFF-score and HGB are outside the calculated range, the expert panel analyses additional information such as the athlete’s whereabouts, journeys, and lab and medical documentation (36).

The ABPS is a value calculated from seven measured haematological parameters to get a combined score that predicts doping more accurately than each parameter alone. It is based on two statistical models (naïve Bayes classifier and support vector machine) and trained on data from both doped and clean (not doped) athletes (37). For clean athletes score values are in the negative range, whereas for athletes who may have doped score values are in the positive range (Table 2) (36,37,38). This score, however, is not sufficient alone to detect doping, because scores above 1 can be found in 1 ‰ of clean male athletes (37).

**Table 2:** Interpretation of abnormal blood profile scores (ABPS)

**ABP*S* value**	**Interpretation**
**<0**	doping not suspected (“clean athlete”)
**0–1**	possible suspicion of doping
**>1**	doping suspected^*^

* Values above 1 can be found in only 1 in 1,000 clean male athletes. ABPS – abnormal blood profile score

### Haematological module: a successful combination of expert review and computer-networked monitoring

The ABP relies on longitudinal computer-networked monitoring that red-flags values or patterns suspicious of doping [under the supervision of the athlete passport management unit (APMU)] and on detailed data review by a panel of experts. In the early days of the ABP, athletes used to blame procedural differences between anti-doping organisations for red-flagged scores, and 5–8 % of such samples were discarded as inadmissible (20). To minimise these issues, WADA has devised ABP operating guidelines that strictly define the procedure of sample collection, transport, storage, and analysis (15). Samples are always to be collected by a trained expert team, stored, and transported in a suitable device at cool temperatures (e.g., 4 °C, freezing not permitted), and delivered to the WADA accredited laboratory for further analysis in time (15, 19, 39). Samples are first tested for potential degradation using the blood stability score (BSS), taking into account transport temperature and time to determine if they are adequate for further analysis (15, 20). To avoid discrepancies between different analysers, samples are always analysed on the same type and model of the analyser (40). During sample collection two blood tubes are taken and the urine sample is divided in two cups. One set (blood tube, urine cup) is labelled as A and analysed immediately, and the other set, labelled as B, is stored for up to 10 years in case additional analyses are necessary (adverse analytical finding, novel methods for doping testing) (1, 41, 42). Laboratory test results are then entered in the anti-doping administration and management system (ADAMS), analysed by the software as described above, and further reviewed by an expert panel if red-flagged. Since the software is more precise if more data are entered into the system, and since athletes are not always tested by the same anti-doping organisation (ADO), WADA has implemented a procedure involving passport custodians, usually within an ADO, who share data obtained by one ADO with other ADOs (15). One passport custodian monitors individual athlete’s data in the ADAMS and ensures that all are entered in their passport (43). Once an atypical passport finding is red-flagged, a three-member expert panel reviews all available data about the athlete (blood donation, menstrual cycle, disease, altitude training, etc.), and decides if the finding is adverse, that is, indicative of doping. Such adverse passport finding is reported to the passport custodian and may be declared an anti-doping rule violation (ADRV) and sanctioned accordingly (15). Athletes can offer different explanations for an atypical passport finding (potential confounders), which are either verified by scientific evidence or not. For example, female athletes often explain a suspicious drop in HGB with heavy menstrual bleeding, but scientific evidence reports little effect of the menstrual cycle on the haematological profile of the ABP (44).

### Steroidal module of the ABP

The steroidal module of ABP was first officially implemented in 2014 to address discrepancies in the detection of anabolic androgen steroids (AAS) (15). It is designed after the haematological module and utilises the same Bayesian adaptive model to examine a series of specific endogenous AAS urine concentrations and ratios (1, 15). The parameters included in longitudinal monitoring are androsterone, testosterone, epitestosterone, etiocholanolone, 5α-androstanediol, and 5β-androstanediol. Calculated ratios include testosterone/epitestosterone (T/E), androsterone/testosterone (A/T), androsterone/etiocholanolone (A/Etio), and 5α-androstanediol/5β-androstanediol (5a-diol/5b-diol) (Table 1). Urine sample collection, transport, analysis, and storage follow the strict procedure given in the ABP Operating Guidelines (15).

Before the module was introduced, anabolic steroid use was detected with the T/E ratio because it is expected to be constant in urine (around 1) (45). In other words, it would increase if testosterone or its analogues are used (45,46,47). Considering individual variations, WADA has set the cut-off value at 4, and all values above are considered suspicious (48). This approach was compromised when one pharmaceutical company developed two products discovered during the Bay Area Laboratory Co-operative (BALCO) scandal (1988–2002) named “The Cream” and “The Clear” (49). “The Cream” is a transdermal combination of testosterone and epitestosterone, whereas “The Clear” is a synthetic anabolic steroid tetrahydrogestrinone that activates testosterone and progesterone receptors. Neither product changes the endogenous T/E ratio (49, 50). In contrast, this ratio is significantly lowered by the *UGT217B* homozygous (*del/del*) polymorphism, found in 80 % of Asian as opposed to only 6.9 % of Caucasian athletes, according to one study (51).

To account for the gene polymorphism, WADA has implemented the steroidal module by adjusting the ABP baseline reference ranges for individual epidemiological characteristics (sex, age, geographical origin, and alcohol consumption) and population reference values with each new entry (52). If the calculated and longitudinally followed T/E ratio falls out of the predicted reference range in athletes 19 years of age (end of puberty) and over, further analyses are required (53).

To account for the substances not affecting the T/E ratio, the steroidal module has included three more parameters, namely the A/T, A/Etio, and 5a-diol/5b-diol ratios. They describe the intrinsic catabolism of testosterone, with the expected drop in the A/Etio and 5a-diol/5b-diol when oral anabolic steroid preparations are used (53, 54).

Finally, since all parameters are influenced by the urine specific gravity, this parameter is also calculated into the steroidal module, as specific gravity below 1.001 points to tampering with substances that dilute urine and lower the concentrations of measured metabolites, which can lower the above ratios (15).

### Current limitations and the future of the ABP

The ABP is designed to track changes in specific parameters to detect artificially induced increases or decreases, which is why the model can sometimes be challenged by physiological and/or pathophysiological aberrations. However, extending parameter limits to account for these aberrations diminishes subtle detection of substance microdosing (e.g., of rHuEPO). While some confounding factors are accounted for in either ABP module (e.g., race, sex, alcohol in the steroidal module, blood donation or bleeding in the haematological module) (15, 21), some are not. For example, plasma volume is a potential confounding factor on which depend HGB and Hct findings, and an adequate replacement method is yet to be included in the ABP. Current research has been looking into indirect markers less dependent on shifts in plasma volume, such as total HGB mass (55,56,57,58), reticulocyte haemoglobin equivalent, immature reticulocyte fraction, and iron metabolism (59,60,61). Because of multiple potential confounders and novel anabolic steroids, every suspicious finding is further analysed with isotope ratio mass spectrometry (IRMS), a method that has shown great sensitivity in detecting exogenous steroids even when the ABP does not red-flag a suspicious finding (62, 63). To address possible urine tampering (e.g., diuretics, substances that change AAS metabolism), new tests to measure steroids in blood before they metabolise are also being evaluated (64). Recent studies are also focusing on detection and quantification of specific doping-related changes at the cellular level (morphology, transcriptomic, proteomics, metabolomics) (65,66,67).

Even though the ABP has its limitations, and more sophisticated direct detection methods are being developed, it remains the key part of the fight against doping (15, 35). Ever since both ABP modules have been implemented, the ABP has revealed the so-called anti-doping rule violations (ADRVs) in 16 % of cases from 2014 to 2020 (68). Now a new, endocrine module is pending implementation. It will monitor changes in measured insulin-like growth factor 1 (IGF-1) and procollagen III peptide (P-III-NP) levels, adjusted for age and sex with the GH-2000 score to detect growth hormone misuse (69,70,71) (Table 1). It still needs to resolve challenges of high intra-individual variability of the included parameters (70, 71).

## CONCLUSION

Since the haematological module was first implemented in 2009, the ABP has made a breakthrough in the fight against doping. This indirect system combining longitudinal monitoring, calculation of individual reference ranges, and review of atypical passport findings by an expert panel has allowed detection of doping even when the exact substance of abuse cannot be identified because as athletes abuse doping to improve their performance their biological parameters change. Even though athletes and their teams sometimes challenge the system, it remains an efficient method of doping detection, whose future looks bright with the new endocrine module and an eye on implementing artificial intelligence.
